# Advances and challenges of the cell-based therapies among diabetic patients

**DOI:** 10.1186/s12967-024-05226-3

**Published:** 2024-05-08

**Authors:** Ramin Raoufinia, Hamid Reza Rahimi, Ehsan Saburi, Meysam Moghbeli

**Affiliations:** 1 Noncommunicable Diseases Research Center, Neyshabur University of Medical Sciences, Neyshabur, Iran; 2 Department of Medical Genetics and Molecular Medicine, School of Medicine, Mashhad University of Medical Sciences, Mashhad, Iran

**Keywords:** Diabetes, Cell therapy, Translplantation, Islet, Immunosuppression

## Abstract

Diabetes mellitus is a significant global public health challenge, with a rising prevalence and associated morbidity and mortality. Cell therapy has evolved over time and holds great potential in diabetes treatment. In the present review, we discussed the recent progresses in cell-based therapies for diabetes that provides an overview of islet and stem cell transplantation technologies used in clinical settings, highlighting their strengths and limitations. We also discussed immunomodulatory strategies employed in cell therapies. Therefore, this review highlights key progresses that pave the way to design transformative treatments to improve the life quality among diabetic patients.

## Background

Diabetes mellitus poses a formidable global public health challenge due to its rapid growing prevalence and associated morbidity, disability, and mortality [1]. According to the International Diabetes Federation, over 537 million adults aged 20–79 had diabetes worldwide in 2021 that is expected to rise to around 783 million cases by 2045 [2]. Obesity, unhealthy diets, physical inactivity as well as genetic and epigenetic predispositions are important risk factors of diabetes [3–5]. Diabetes is typically classified into type 1 diabetes mellitus (T1DM), gestational diabetes mellitus (GDM), and type 2 diabetes mellitus (T2DM) [2]. T1DM primarily arises from autoimmune-related damage of insulin-secreting beta cells, resulting in severe hyperglycemia and ketoacidosis [6]. In contrast, T2DM generally has a more gradual onset characterized by insulin resistance along with diminished compensatory insulin secretion from pancreatic beta cell dysfunction [7]. Diabetes is associated with macrovascular complications such as heart disease and stroke, as well as microvascular issues in eyes, kidneys, and nervous system [8]. Cancer is also a leading cause of diabetes-related death, and dementia-associated mortality has risen in recent decades [9–12]. Cell therapy involves transferring autologous or allogenic cellular material into patients [13]. The global market size of cell therapy is estimated to grow from $9.5 billion in 2021 to $23 billion by 2028 [14]. It combines stem and non-stem cell therapies consisting of unicellular or multicellular preparations. Cell therapies typically use autologous or allogenic cells via injection and infusion [15]. In the present review, we discussed the recent advances in cell-based therapy of diabetes, from foundational islet transplantation to regenerative strategies to highlight key developments that improve the effective treatments for diabetic patients.

### Cell replacement therapy for diabetes

Pancreatic transplantation was firstly used in 1966 to treat type 1 diabetes using whole organ transplants. During the 1970s–80s, segmental pancreatic grafts were combined with techniques to divert digestive secretions away from transplanted cells. Three main techniques emerged; simultaneous pancreas-kidney transplants, pancreas transplants following kidney transplants, and pancreatic transplants. International collaboration on tracking outcomes began in 1980 with the formation of several pancreatic transplant registries and associations. However, whole organ transplantation was faced with several challenges including organ rejection, vascular complications, limited organ availability, and the effects of lifelong immunosuppression [16, 17]. Islet cell transplantation was explored as an alternative, however isolating and transplanting pancreatic islets proved difficult due to donor availability, rejection, and immunosuppression side effects. Recent research has focused on stem cell sources that could reconstitute immune tolerance and preserve beta cell function such as mesenchymal stem cells, bone marrow cells, and embryonic stem cells [18]. A novel stem cell therapy called VX-880 was developed using proprietary technology to grow insulin-producing beta cells from allogeneic stem cells. Clinical trials began in 2021 after FDA approval to deliver the cells intrahepatically under immune suppression. A second approach called VX-264 encapsulates the same cells, avoiding immunosuppression but requiring surgical implantation [17]. In 2023, FDA approved the first allogeneic pancreatic islet cell therapy called Lantidra for adults with type 1 diabetes experiencing severe hypoglycemia. Approval was based on two studies where 21–30% of participants no longer required insulin one year post-treatment, with benefits lasting over five years in some cases. However, this treatment have mild and serious adverse events that are associated with treatment dose and the methods of islet cell infusion [19, 20].

### Emerging strategies for cell delivery via microencapsulation and biological devices in clinical trials

#### Alginate capsules as cell delivery systems

A seminal investigation conducted in 1994 demonstrated the successful transplantation of alginate-encapsulated islets into the peritoneum of kidney transplant patients who were receiving immunosuppression therapy. Remarkably, these patients achieved insulin independence for up to nine months [21]. However, subsequent trials conducted without immunosuppression yielded inconsistent outcomes. In a study conducted in 2006, islets were encapsulated in triple-layer alginate capsules and implanted intraperitoneally in type 1 diabetes (T1D) patients. There was a positive correlation between the encapsulation and insulin production that reduced exogenous insulin requirements during one year. Despite this progress, the entry of cytokines remained a potential concern [22]. Another study employed the single-layer barium-alginate capsules that sustained insulin production for up to 2.5 years [23]. It has been reported that the microneedle, comprising a calcium alginate frame with polydopamine-coated poly-lactic-co-glycolic acid microspheres encapsulating insulin, enables light-triggered insulin release. Microneedle provided a suitable insulin dose to maintain blood glucose levels in line with daily fluctuations. These results established the efficacy and safety of the developed microneedle for diabetes treatment [24]. Another therapeutic approach explored the encapsulation of pancreatic islets with mesenchymal stem cells (MSCs) and decellularized pancreatic extracellular matrix (ECM). ECM derived from the pancreas supported islet cell growth and maintenance to enhance insulin expression [25]. Sodium alginate and hyaluronic acid were incorporated due to their roles in collagen production, wound healing, and physical crosslinking. The 3D porous membranes allowed optimal water and oxygen transfer while diverting excess exudate from diabetic wounds. Hydrogel accelerated re-epithelization, while decreased inflammation, indicating potential as the diabetic wound dressings [26]. Additionally, the incorporation of specific ECM components, such as collagen IV and RGD, into alginate-based microcapsules significantly improved the survival, insulin secretion, and longevity of microencapsulated islets [27].

### Encaptra® device from ViaCyte

In contrast to microencapsulation techniques, ViaCyte developed a semipermeable pouch method named Encaptra, which contains pancreatic precursor cells derived from the embryonic stem cells [28]. In the initial trial conducted in 2014, the “VC-01” device was implanted in T1D individuals without the use of immunosuppression [29]. The trial confirmed the safety of the device; however, the occurrence of hypoxia induced cellular necrosis [30]. The device was modified as “VC-02” with larger pores, and two trials (NCT03162926, NCT03163511) demonstrated promising outcomes, including increased fasting C-peptide levels and a 20% reduction in insulin requirements during one year in the majority of participants [31]. In order to eliminate the necessity for immunosuppressants, ViaCyte collaborated with Gore to develop an expanded polytetrafluoroethylene (ePTFE) device with both immuno-isolating and pro-angiogenic properties [32]. This device (NCT04678557) aimed to prevent immune cell attachment and T-cell activation [33]. Additionally, ViaCyte is exploring the integration of CRISPR technology to modify stem cells, specifically by eliminating β2-microglobulin expression and PD-L1 up regulation. It is hypothesized that these genetic modifications will further hinder immune cell attachment and T-cell activation [30, 34].

### Semipermeable device from Semma therapeutics

Semma Therapeutics, which has been acquired by Vertex, pioneered the utilization of differentiated stem cell-derived islet cell clusters in clinical trials. Semma houses these cells between two semipermeable polyvinylidene fluoride membranes and is designed for subcutaneous implantation (NCT04786262) [31, 35]. Vertex reported a significant breakthrough by infusing differentiated beta cells via the portal vein in a participant who was receiving immunosuppressants. This approach led to substantial C-peptide production and improved glycemic control during 90 days [36].

### βAir device from Beta O2

Beta O2’s innovative βAir device utilizes an alginate-PTFE membrane complex to encapsulate islets, providing partial immunoisolation while ensuring a continuous supply of oxygen, which is crucial for optimal islet function [37, 38]. The βAir device that was seeded with human islets was subcutaneously implanted in T1D individuals (NCT02064309). Although, low insulin levels were produced for up to eight weeks, there was not any reduction in the required exogenous insulin [37]. While, increasing the number of islets could potentially enhance their function, it is important to note that the continuous reliance on oxygen poses a risk of infection, despite efforts to optimize the survival of encapsulated islets [39, 40].

### Cell pouch™ device from Sernova

Sernova has developed the Cell Pouch device, which offers pre-vascularized polypropylene chambers for islet transplantation without the need for immunoprotection. The device consists of multiple cylindrical chambers that are prefilled with PTFE plugs, which are then removed after implantation to create the empty space [41]. In a 2012 trial (NCT01652911), islets were placed in the vascularized pouches of three recipients who were also receiving immunosuppression that resulted in a transient increase in C-peptide levels [41]. In a 2018 trial (NCT03513939), immunosuppression was administered after implantation and islet introduction. This trial reported sustained C-peptide production for up to nine months in two recipients, along with improved glycemic control [42]. Regarding the limitations of immunosuppression, Sernova is exploring the possibility of encapsulating islets in hydrogel as an alternative approach [43].

### Shielded living therapeutics™ from Sigilon Therapeutics

Sigilon has developed the Shielded Living Therapeutics sphere, which consists of cell clusters enclosed within an alginate-TMTD coating [44]. Preclinical studies demonstrated that murine islet transplants encapsulated within these spheres maintained normoglycemia for a period of six months [45]. In a 2020 trial conducted for hemophilia (NCT04541628), the spheres were evaluated for their ability to express Factor VIII [46]. However, the trial was paused due to the development of antibodies in the third recipient receiving the highest cell doses. While, preclinical studies have shown promising efficacy, there are safety concerns regarding the TMTD coating that need to be addressed before these spheres can be used for human islet transplantation as a treatment for diabetes [31]. Emerging technologies have been investigated in clinical trials for delivering insulin-producing islets or stem cell-derived beta cells via microencapsulation or use of implantable biological devices (Table 1). Optimizing encapsulation and developing alternative implantable devices moves the field toward delivering safe and effective islet replacement without chronic immunosuppression dependency that represented an important new frontier for the cell-based treatment of diabetes. However, continued refining will be required to fully realize this promising vision and using these preclinical concepts in clinic.

### Immunoengineering strategies: biomaterials for modulating immune responses

**Fig. 1:**
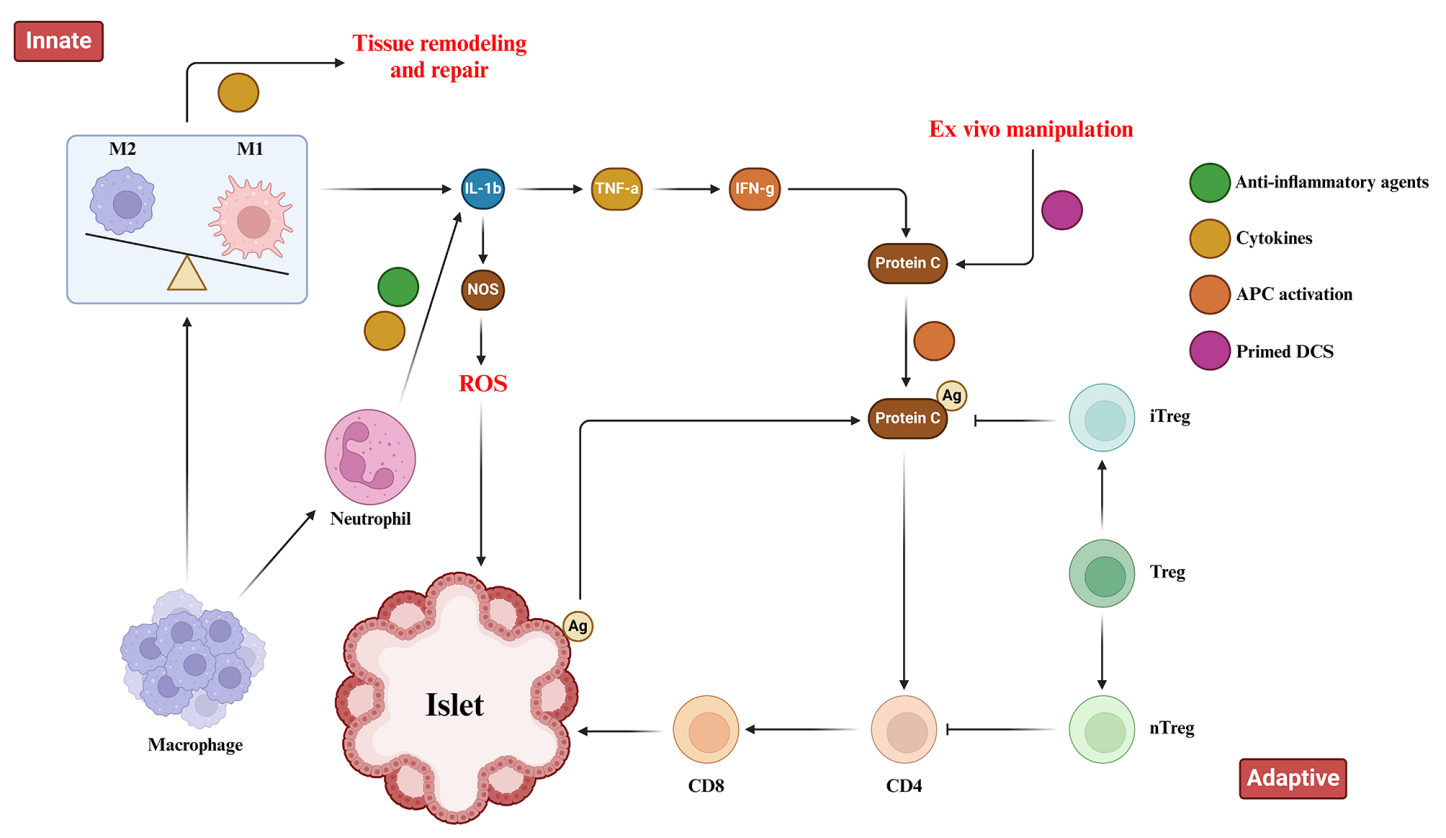
Immune Responses toward pancreatic islets following transplantation. This figure illustrates the immune responses, including the innate and adaptive immunity that are triggered upon pancreatic islet transplantation. Immune response begins with the activation of tissue macrophages and neutrophils in response to injury. Subsequent, release of inflammatory cytokines stimulates antigen-presenting cells (APCs), CD4 + T cells, CD8 + T cells, and cytotoxic T lymphocytes to orchestrate the immune response

Islet encapsulation aims to prevent immune responses toward transplant antigens. However, foreign body response (FBR) against biomaterials induces inflammation around encapsulated islets that obstructs oxygen/nutrient access and causes graft failure [31]. Extensive research revealed biomaterial properties profoundly influence FBR severity, with high purity/biocompatibility moderating inflammation [47]. Deeper understanding of biomaterial immunobiology enabled developing immune-modulating constructs to steer host interactions. By altering topology/chemistry to hinder nonspecific binding and cell adhesion, these “immune-evasive biomaterials” intended to attenuate xenograft rejection at inception [44]. Both innate and adaptive immune responses have crucial roles in the context of pancreatic islet transplantation. These responses encompass the activation of tissue macrophages and neutrophils following injury, leading to the release of inflammatory cytokines that subsequently activate antigen-presenting cells (APCs), CD8 + T cells, CD4 + T cells, and cytotoxic T lymphocytes (Fig. 1). Zwitterionic polymers conferred anti-fouling attributes but crosslinking limitations constrained their application [48]. Novel mild zwitterionization introduced alginate modifications that prolonged prevention of fibrotic overgrowth by mitigating initial responses [49–51]. The prevention of graft rejection following islet cell transplantation necessitates the systemic administration of immunosuppressive agents. While, these agents effectively suppress immune responses, their continuous use exposes patients to an increased risk of infection and cancer. To mitigate these concerns, an alternative approach involving the localized delivery of immunosuppressants at the transplantation site has emerged. This localized delivery system offers several advantages, including targeted drug delivery, reduced systemic exposure, and potentially reduces the immunosuppressants doses [52]. Polymeric carriers dispersed cyclosporine A continuously at the graft site to dynamically tamp down proinflammatory cascades and T-cell activation [53, 54]. TGF-β/IL-10 co-delivery at the microencapsulation interface hindered innate antigen presentation, obstructing adaptive response priming [55, 56]. Regulatory T-cells emerged as the potent immunomodulators when coated on islets to improve insulin production in vitro [57]. Similarly, recombinant Jagged-1 surface patterning increased regulatory lymphocytes in vitro while enhancing glycemic oversight in vivo [58]. Targeting proinflammatory effector T-cells or presenting their Fas ligand death receptor improved long-term viability when combined with rapamycin prophylaxis [52, 59]. Immobilizing thrombomodulin or urokinase mitigated local inflammation, with the latter conferring lifelong xenotransplant survival [60]. Peptides recognizing IL-1 receptors provided robust protection from destabilizing proinflammatory cytokines [61]. Leukemia inhibiting factor improved islet performance over polyethylene glycol encapsulation alone by inducing regulatory T-cell lineages [62]. Silk scaffolds facilitated IL-4/dexamethasone emancipation that meaningfully decreased immune reactions to grafts [63]. Therefore, the localized delivery of immunosuppressants at the transplantation site represents a promising strategy for islet cell transplantation. Compared to systemic administration, local delivery can achieve targeted immune modulation only at the graft location while reducing drug exposure throughout the body. This localized approach aims to sufficiently suppress the immune response to prevent rejection, while limiting negative side effects that may occur from systemic immunosuppression. A variety of biomaterials and surface modification strategies have been developed and investigated for the local delivery of immunosuppressive agents and immunomodulatory cytokines [64–66]. Understanding how biomaterial properties influence the immune response is critical to design biomaterials that can modulate inflammation and improve islet graft survival through localized immunomodulation.

### Cell-based therapy through the integration of additive manufacturing techniques

Additive manufacturing utilizes computer modeling to fabricate complex 3D structures on-site with minimal post-processing. Common methods for the biomedical application are fused filament fabrication (FFF), stereolithography (SLA), and bioprinting [67]. FFF is a layer-by-layer technique that extrudes heated thermoplastics [68]. Commonly used feedstocks include acrylonitrile butadiene styrene (ABS) and polylactic acid (PLA). Other thermoplastics that have been utilized with FDM include thermoplastic polyurethane (TPU), polycarbonate (PC), polystyrene (PS), polyetherimide (PEI), polycaprolactone (PCL), polyaryletherketone (PAEK), and polyetheretherketone (PEEK), with the latter demonstrating high strength and heat tolerance. A major advantage of FDM is its ability to fabricate multi-material objects through continuous printing and alteration of the build material. In addition to typical polymers like PC and polystyrene (PS), FDM can print composites reinforced with glass, metals, ceramics, and bioresorbable polymers via integration of the constituent powders with a binding matrix. This enables enhanced control over the experimental component fabrication. While, ceramic and metal filaments traditionally contain the corresponding powder mixed with a binder, FDM provides versatility in the functional prototype construction from a wide range of thermoplastic feedstocks using precise and additive layer manufacture [68–72]. It provides geometric reproducibility and reduced variability compared to traditional techniques. FFF prints served as scaffolds for the transplanted cells [67]. However, minimum feature size is limited to ?∼ 250 μm by nozzle diameter [68]. SLA employs light-curable liquid resins and achieves higher 50–150 μm resolution than FFF but with restricted material choices. Bone grafts and surgical guides are common applications [67]. Incorporating biomaterials like hydroxyapatite has expanded utility, though processing is required to mitigate cytotoxicity. Additive manufacturing can address limitations in oxygen transport, cell/material placement control and vasculature formation, and clinically translatable insulin-secreting implants [67]. Therefore, additive manufacturing technologies have the potential to enhance various aspects of the cell-based transplant design, from improving nutrient transport through optimized implant geometry to achieving precision integration of therapeutic agents (Table 2).

### Enhancing nutrient transport through optimization of implant geometry

Tissue engineering for the islet transplantation requires maximizing nutrient transport [73, 74]. Traditional scaffold fabrication introduces macroporosity but lacks precision that results in inflammation [67]. Cell encapsulation provides immunoprotection by limiting interactions between transplanted cells and the host immune system. However, this protective barrier also poses challenges for the efficient transport of essential nutrients, including oxygen, to the encapsulated cells. Modifying the geometries of encapsulation devices using conventional methods to enhance oxygen delivery has proven to be inconsistently challenging [67], so that novel approaches are required to address these challenges. Additive manufacturing allows customizing biomaterial scaffolds with defined geometries and micropore sizes to improve transport [75–79]. The 3D printed PLA scaffolds with islets have successful vascularization and cellular survival after subcutaneous transplantation [80, 81]. Interlocking toroidal hydrogel-elastomer constructs also increased surface area and cell viability [82–84].

### Enhancing vascularization and engraftment

Rich host vascularization of transplant devices is essential to support long-term islet survival through efficient nutrient delivery and insulin kinetics. Early platforms modified bulk material properties to promote vessel infiltration and anastomoses [85–89]. Additive manufacturing can further optimize microscale geometry to both accelerate host vessel connections and control intra-device vasculature homogeneity beyond traditional fabrication. Initial work reproduced macroscale vessels but scales were diverged from cell-based therapies [73, 90–92]. Leveraging Additive manufacturing designed structures guided vessel formation in vitro and in vivo [80, 89, 93]. Shifting to bioprinting complex branching conduits in supportive hydrogels facilitated clinical translation for diverse cell therapies [94–98]. Researchers focused on developing a 3D scaffold platform to improve the transplantation outcomes of islet cells in T1D. The scaffold featured a heparinized surface and immobilized vascular endothelial growth factor (VEGF) to enhance vascularization. Scaffold effectively promoted angiogenesis and facilitated the growth of new blood vessels. Additionally, encapsulated islets within the scaffold had functional responses to glucose stimuli. These findings suggested that the developed scaffold platform holds potential for successful extra-hepatic islet transplantation, offering new possibilities for T1D treatment [99]. Research on vascularization of islets via additive manufacturing techniques has primarily focused on the fundamental discoveries. In one study, engineered pseudo islets (EPIs) were created by combining the mouse insulin-secreting beta cells with rat heart microvascular endothelial cells. EPIs demonstrated extensive outgrowth of capillaries into the surrounding matrix. Although, EPIs containing both cell types that underwent capillarization maintained viability and function over time in culture, non-vascularized EPIs lacking endothelial cells could not sustain viability or functionality long-term. This supported the potential for inducing angiogenesis within bioengineered islet constructs. Future work may combine patient-specific stem cell-derived human beta cells with endothelial cells using this approach to promote long-term graft survival for treating type 1 diabetes [98]. While, large-scale 3D printed vascularized structures are currently limited for the islet transplantation, advancements in leveraging additive manufacturing for the optimization vascularization conditions through the pore sizes and material choices, may facilitate translation to β-cell therapy in type 1 diabetes.

### Precision placement of cells and matrix for enhanced control

Beyond distributing biomaterials, additive manufacturing enables micro-level cell and protein control. For islet transplantation, optimal cellular distribution and supportive extracellular matrix niche reduce rapid dysfunction and apoptosis [100–102]. Traditional techniques heterogeneously load cells after fabrication or struggle with incomplete encapsulation [103, 104]. Bioprinting allows in situ encapsulation and printing of multiple cell types and matrix components while dictating 3D placement and dimensions [105, 106]. Islet transplant research prints hydrogel-encapsulated clusters surrounded by supportive cells and doped with immune modulators to improve the transplant environment [107]. Progress in bioprinting offers consistency and defines physical/chemical graft properties beyond traditional fabrication.

### Achieving controlled integration of therapeutic agents for enhanced efficacy

In addition to the cell and matrix placement, additive manufacturing enables precision therapeutic integration. Incorporating therapeutics aims to recapitulate the in vivo environment through angiogenesis, islet health promotion, and immunomodulation [67, 108]. Growth factors promote vessel formation and insulin secretion while decrease apoptosis [108–111]. Local immunomodulators regulate the immune system in a specific site of the body. They decrease inflammation and promote the successful integration of transplanted cells or tissues by minimizing the need for widespread immune suppression in whole body [67]. Traditional homogeneous delivery methods restrict the ability to customize the spatial distribution of substances and pose a risk of harmful effects on transplants or hosts [112]. The use of discreet gradients in bioprinting can offer precise physiological signals. By combining traditional drug release methods with AM, it becomes possible to create tissues that exhibit distinct therapeutic localization. Bioprinted composites have the ability to release factors with gradients throughout the entire construct that enables a more comprehensive and targeted approach in tissue engineering [112–114].

### Cell based gene therapy

Gene therapy holds great promise for diabetes management, offering innovative approaches to deliver and manipulate the insulin gene in various tissues. Viral methods, such as lentivirus, adenovirus, and adeno-associated virus (AAV), along with non-viral techniques like liposomes and naked DNA, have been utilized to deliver the insulin gene to target tissues [115]. This section aims to provide an overview of important studies in the field of gene therapy for diabetes management, emphasizing advancements in insulin gene delivery and manipulation (Table 3).

### Enteroendocrine K-cells and pancreatic β-cells

Enteroendocrine K-cells in the intestines and pancreatic β-cells share similarities in their production of glucose-dependent insulinotropic polypeptide (GIP) and their regulatory mechanisms. Understanding these similarities offers insights into T2D management and improving glucose homeostasis. However, attempts to reverse diabetes effectively through K-cell transplantation have been unsuccessful. Nevertheless, research on gene editing techniques has shown promising results in management of the diabetes mellitus [116, 117]. AAV vectors have been employed to co-express insulin and glucokinase genes in skeletal muscles, demonstrating long-term effectiveness in achieving normo-glycemia without exogenous insulin [118, 119].

### Gene editing techniques

Gene editing techniques using AAV vectors effectively improved normo-glycemia in animal models. Co-expression of insulin and glucokinase in transgenic mice increased glucose absorption and regulated insulin production. Duodenal homeobox 1 (PDX1) gene transfer via AAV2 in a humanized liver mouse model also led to insulin secretion and glycemic control [120]. Adenovirus-mediated transfection of hepatic cells with neurogenin 3 (NGN3) resulted in insulin production and trans-differentiation of oval cell populations [121, 122]. Targeting specific promoters in liver cells such as phosphoenolpyruvate carboxykinase (PEPCK), glucose 6-phosphatase (G6Pase), albumin, and insulin-like growth factor binding protein-1 (IGFBP-1) enhanced hepatic insulin gene therapy [123, 124]. AAV-mediated overexpression of SIRT1 reduced inflammation, hypoxia, apoptosis and improved neural function in the retina of diabetic db/db mice [125]. Another study developed a plasmid expressing a single-strand insulin analogue for intramuscular injection using a specialized gene delivery technique. A single administration provided sustained insulin expression for 1.5 months and effectively regulated blood glucose levels without immune responses or tissue damage in diabetic mice.

### Non-viral gene delivery methods

Non-viral approaches have also key roles in achieving glycemic control. The combination of insulin fragments with DNA plasmid, administered via intravenous injection improved normo-glycemia for extended periods. DNA transposon facilitated gene integration into the host chromosome that addressed the short-term liver expression. Additionally, the co-injection of DNA plasmid containing insulin with furin significantly enhanced insulin production within muscles [126]. Non-viral plasmids were engineered to carry proinsulin and pancreatic regenerating genes to ameliorate streptozotocin-induced T1DM [127]. The pVAX plasmid vectors prolonged therapeutic effects in achieving normo-glycemia without the need for further treatment [127]. Bioreducible cationic polymers, such as poly-(cystamine bisacrylamide-diamino hexane) (p(CBA-DAH)), have been employed to deliver RAE-1 to pancreatic islets, resulting in improved insulin levels [128]. Furthermore, ex vivo gene transfer and autologous grafts have shown promising outcomes in animal models. The introduction of the human insulin gene into pancreatic or liver cells followed by autologous grafts improved insulin secretion, glycemic control, and alleviated the diabetic complications in pigs. However, gene silencing eventually occurred, necessitating a deeper understanding of the underlying mechanisms [128, 129].

### Stem cell based therapy in diabetes

**Fig. 2:**
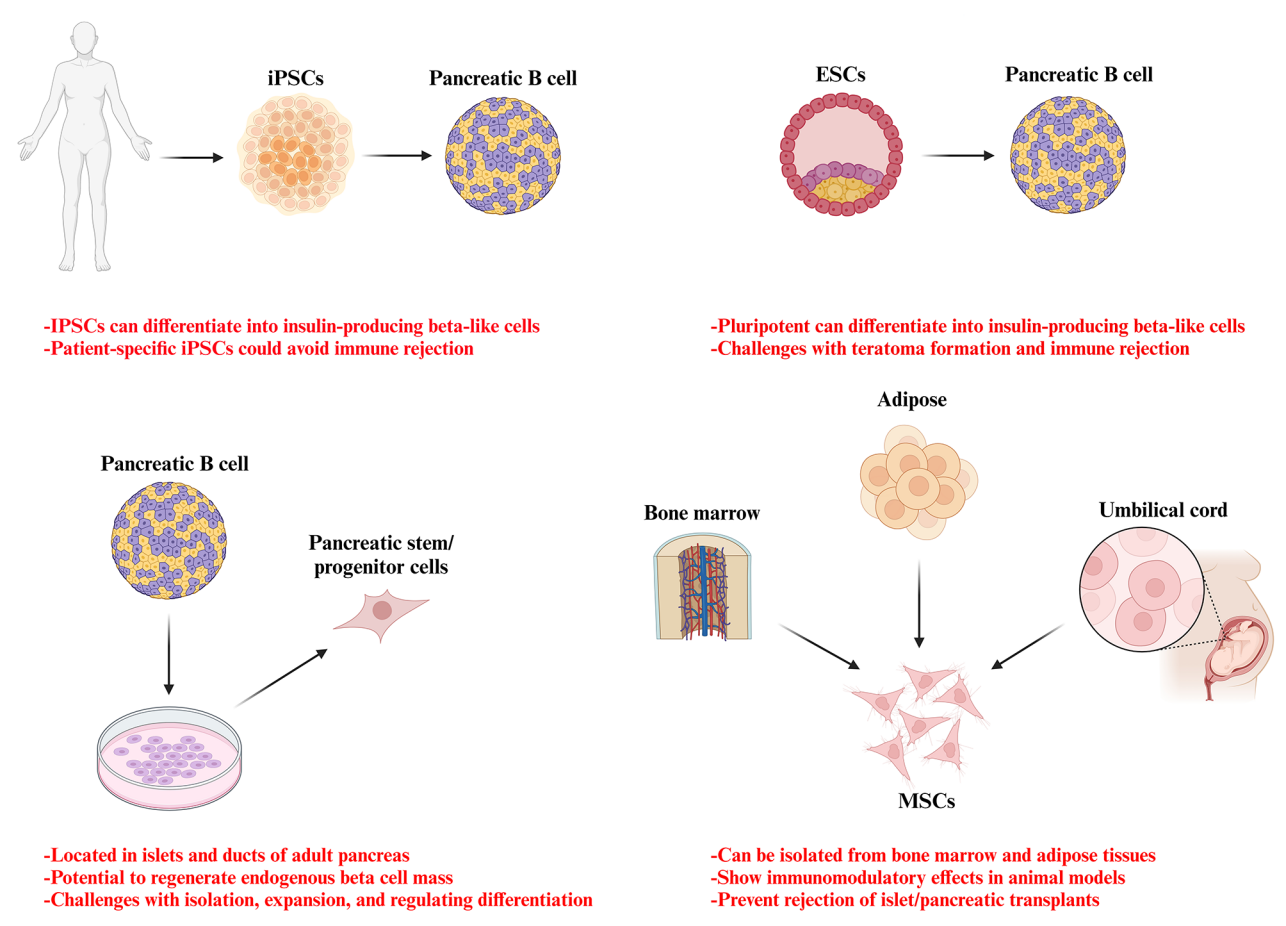
Potential stem cell sources for the treatment of diabetes

Efforts are ongoing to develop standardized processes for donor and recipient selection/allocation to increase pancreas utilization [130–133]. Techniques for isolating pancreatic islets are being optimized to become more standardized and consistent. Noninvasive imaging technologies allow the monitoring of the transplanted islets without surgery [134, 135]. Biomarkers could also evaluate how immunomodulation strategies are working [136–138]. Researchers are also exploring alternative transplant sites in the body beyond just the liver, to see if the other locations may better support islet graft survival and function. Together, these areas of refinement aim to improve the safety and reliability of islet transplantation procedures as a potential therapy for diabetes [139]. Bioengineering approaches are being developed to optimize the islet transplantation microenvironment using biomaterials which enhance islet engraftment and function through engineered extracellular niches [140, 141]. For example, encapsulation techniques aim to protect pancreatic islets against immune reponse by enclosing them within semipermeable hydrogel polymer capsules [142, 143]. This localized immunoisolation strategy utilizes biomaterials like alginate to create a physical barrier preventing immune cell contact while still allowing nutrient and oxygen diffusion. Researchers concurrently seek alternative unlimited cellular sources to address limited islet availability. Mesenchymal stem cells possess immunomodulatory properties and their adjuvant delivery, either early in disease onset or simultaneously with islet transplantation, has shown promising signs of improving outcomes in preclinical investigations. By dampening inflammatory responses and favoring regenerative processes, stem cells may help to establish a more tolerogenic transplant environment. These bioengineering and cell therapy approaches offer potential pathways towards eliminating the exogenous insulin requirement [144, 145]. A variety of stem cell types have therapeutic potential for diabetes (Fig. 2). Pluripotent stem cells possess immense promise for overcoming the limitations of islet transplantation. Human embryonic stem cells and induced pluripotent stem cells are especially attractive candidates due to their unique ability to both self-renew indefinitely and differentiate into any cell type. This makes them an ideal source of replacement pancreatic beta cells. Significant research effort across academic and industrial laboratories has led to advancement in differentiation protocols that can convert pluripotent stem cells into functional beta-like cells in vitro. However, establishing consistent, well-characterized cellular production methods that comply with stringent safety and efficacy standards remains a priority for clinical translation. Ongoing work aims to generate therapeutic stem cell-derived beta cell replacements exhibiting stable, glucose-responsive insulin secretion comparable to primary islets. Although, technological and regulatory hurdles still must be cleared, pluripotent stem cells have the greatest potential to finally solve the problem of limited cell availability and provide an unlimited source of transplantable tissue suitable for widespread treatment of diabetes [145–148]. There are currently six registered clinical trials evaluating the use of human pluripotent stem cells for the T1D treatment. All trials except one use PEC-01 cells, which consist of a mixture of pancreatic endoderm and polyhormonal cell population derived from CyT49 stem cells that are fully committed to endocrine differentiation upon implantation [149]. The initial trial implanted PEC-01 cells within an encapsulation device, hypothesizing no need for immunosuppression. While, well-tolerated with minor adverse effects, insufficient engraftment occurred due to foreign body responses that eliminated the cells [150]. The trial transitioned in 2017 to use an open encapsulation device that required immunosuppression. Subcutaneous engraftment, differentiation of cells into islet-like clusters, and glucose-responsive insulin production provided the first evidence that pancreatic progenitor cells can survive, mature, and function as the endocrine cells in humans. Potential benefits on stimulated C-peptide levels and glycemic control were observed in one patient [151, 152]. Two reports in late 2021 described results in 17 patients receiving PEC-01 cells in an open device. Engraftment and insulin expression occurred in the majority, glucose-responsive secretion in over one-third, and various glycemic improvements were observed at six months. Explanted tissues contained heterogeneous pancreatic compositions including mature beta cells, with no teratoma formation and mild adverse effects related to surgery/immunosuppression. VX-880 uses fully differentiated insulin-producing stem cell-derived islet cells in phase 1/2 trial evaluating portal infusion and different doses requiring immunosuppression. Preliminary results suggest early engraftment and insulin secretion. The manin challenge was controlling immune rejection without systemic immunosuppression [149]. Several strategies are being explored to address the challenges of immune rejection in stem cell therapies for diabetes. They include generating stem cell lines that are universally compatible through HLA silencing, developing milder regimens of immunosuppression, and refining encapsulation and containment approaches to protect transplanted cells toward immune response. Establishing standardized stem cell banks is also an area of investigation [153, 154]. Xenotransplantation using gene-edited porcine islets remains an exciting avenue of research given advances to improve engraftment and reduce immunogenicity in preclinical studies [155]. Novel approaches continue to emerge as well, such as decellularization techniques, 3D bioprinting of tissue constructs, and creating interspecies chimeras. Rapid evolution of cell-based therapies across both academic and commercial sectors is promising to restore normoglycemic control in diabetic cases. Refinement of existing methods and development of new strategies hold potential to perform a safe and effective cell replacement without reliance on systemic immunosuppression. Stem cell and regenerative therapies may ultimately manage diabetes through restored endogenous insulin production [156]. Recently a meta analysis evaluated the safety and efficacy of MSC-based therapy for diabetes in humans. This comprehensive analysis was conducted on 262 patients across six trials that met the inclusion criteria within the last five years. The results reveal that treatment with MSCs significantly reduced the dosage of anti-diabetic drugs over a 12-months. Following treatment, HbAc1 levels decreased by an average of 32%, fasting blood glucose levels decreased by an average of 45%, and C-peptide levels showed a decrease of 38% in two trials and an increase of 36% in four trials. Notably, no severe adverse events were reported across all trials. Therefore, it can be concluded that MSC therapy for type 2 diabetes is safe and effective [157].

### Advances in islet transplantation and stem cell-derived Beta cells

Limited number of the islet transplantation donors highlights the importance of cell therapy in diabetes. Although, higher islet numbers from multiple donors increase the success, limited pancreas availability restricts widespread use [158]. Using multiple donors also increases rejection risk, while isolation of the islets can cause tissue damage [159]. To overcome these challenges, researchers have explored the differentiation of stem cells into beta cells in vitro to generate an unlimited supply of insulin-producing cells with standardized and characterized products. Genetic engineering techniques have also been investigated to confer advantages such as stress resistance or immune evasion [158]. ViaCyte has developed a stem cell-derived pancreatic progenitor called PEC-01, which has the ability to mature into endocrine cells in rodent models. To protect the transplanted cells from immune response, retrieval encapsulation devices were also created [160–162]. In an initial human clinical trial conducted in 2014 (NCT02239354), the Encaptra device was utilized with the aim of providing complete immunoprotection of transplanted cells through the use of a cell-impermeable membrane. Although, the PEC-Encap product showed reliable tolerance and minimal adverse effects, the trial was stopped due to the inadequate engraftment of functional products. While, a few endocrine cells were observed, fibrosis around the capsule led to graft loss and supression of the insulin secretion. To address this challenge, a more recent development called the PEC-Direct device was introduced, which featured openings in the membrane to facilitate vascularization, thereby improving nutrient exchange and supporting cell viability. However, since host cells could infiltrate the device, immunosuppression was necessary following the transplantation [163–165]. Protocols were developed to generate clusters of stem cell-derived beta cells that secreted glucose-responsive insulin. These clusters, referred to SC-islets, also contained other endocrine cells, including glucagon-producing cells. SC-islets improved glycemic control in diabetic mice and nonhuman primates [146, 166–168]. In a trial conducted in 2017 (NCT03163511), the transplantation of progenitor cells resulted in the maturation of endocrine cells, and glucose-responsive C-peptide secretion was observed 6–9 months post-transplantation. Notably, the majority of these mature endocrine cells exhibited glucagon-positive characteristics. The porous regions housing the endocrine cells allowed for the infiltration of host vessels to facilitate vascularization. However, non-cellular regions were isolated by the presence of fibrosis [164, 165]. Although, there was not a sufficient levels of circulating C-peptide in these trials, the findings underscored the significance of promoting vascularization and minimizing fibrotic reactions [164, 169]. Vertex conducted a human trial in 2021 (NCT04786262) involving the transplantation of half-dose VX-880 cells (SC-islets) without a device to avoid previous problems, which necessitated immunosuppression. Preliminary results reported improved glycemic control, although it took longer to achieve the same outcome compared to rodent models [158]. Overall, progresses in islet transplantation and stem cell-derived beta cells pave the way for overcoming the limitations of traditional approaches. Further research and refinements are also required to achieve consistent and clinically significant outcomes in the treatment of diabetes.

### Chalenges and limitations

Cell-based therapies have been significantly progressed for diabetes; however, there are still several challenges that need to be overcome. Clinical trials investigating encapsulation devices and islet transplantation techniques have provided valuable insights but face several obstacles including oxygenation, host immune responses, and insufficient long-term engraftment success. Immunoengineering of biomaterials and additive manufacturing for the development of 3D islet structures aim to modulate inflammation and promote graft revascularization. Nevertheless, achieving consistent normalization of blood glucose levels without exogenous insulin remains a challenge in human studies. In the field of gene therapy and stem cell differentiation, research focuses on genetically-modified or progenitor-derived insulin-secreting β-like cells to optimize protocols that ensure safety and functionality. The main challenge is to establish stable and functional cells capable of permanently restoring normoglycemia without the need for external intervention. One major barrier is the immune response, which targets allogeneic and xenogeneic islet grafts. Although, local immunotherapy minimizes the systemic effects, evading graft destruction through biomaterials without the requirement of immune suppression remains a significant challenge. The translation of precision 3D islet constructs and genetically reprogrammed cells also necessitates scalable manufacturing processes to ensure consistent function and long-term safety across batches. When critically appraising progress in the field of cell-based diabetes treatments, it is imperative to consider the regulatory, ethical, economic, and safety factors that shape translational applications. At the regulatory level, oversight bodies play a pivotal role in establishing standards to ensure patient welfare while enabling therapeutic innovation. FDA oversees clinical trials and product approvals in the United States (US), while in Europe the EMA provides parallel regulatory guidance. Within the US, organizations like the United Network for Organ Sharing (UNOS) and Organ Procurement and Transplantation Network (OPTN) govern organ and cell allocation protocols [17, 170]. However, as regenerative approaches diverge from traditional organ transplantation, regulatory pathways require ongoing harmonization between the agencies and jurisdictions. Continual dialogue between researchers, oversight boards, and policymakers will be crucial to streamline guidelines in a patient-centric manner that balances safety, efficacy, and timely access to cutting-edge therapies. For instance, as stem cell-derived beta cells and 3D bioprinted tissue constructs emerge, traditional drug and device frameworks may not adequately address product characterization and manufacturing complexities for these advanced therapeutic products [67]. Within clinics, maintaining compliance with evolving regulations impacts research directives and ultimately patients’ access to the novel treatments. Addressing informed consent, clinical trial design, and privacy protections for sensitive health data are also paramount from an ethical perspective [128, 129]. Autonomy and agency of research participants in decision-making related to experimental therapies demand prudency. Equitable accessibility of new treatment options also warrants attention to avoid certain populations facing undue barriers. Cell sourcing presents ethical issues depending on derivation from embryonic, fetal or adult tissues. Logistical matters like shipping and processing stem cell-derived islets prior to transplantation necessitate scrutiny. Tumorigenic potential of the undifferentiated pluripotent stem cells should be optimized through rigorous preclinical testing. Transitioning therapies between animal and early human investigations necessitates well-characterized cellular products showing consistent safety and glucose-responsive insulin secretion profiles comparable to pancreatic islets. Long-term animal model data substantiating lack of malignant transformation following transplantation aids allaying ethical safety concerns as the therapies progress clinically. Researchers carefully screen new concepts to prevent side effects in participants while pursuing curative goals. In terms of economic costs, islet and stem cell transplant procedures remain prohibitively expensive for broad applicability despite promising clinical signals. The field requires sustained study to validate techniques, track long-term outcomes, assess healthcare costs offsets from mitigating diabetes’ debilitating complications, and establish cost-benefit ratios for national reimbursement paradigms. Public-private partnerships may accelerate large, interventional trials and longitudinal research to precisely quantify the cellular therapies’ safety profiles and real-world efficacies compared to intensive management versus costs of intensive diabetes care. Ongoing developments like 3D bioprinting offer catalytic manufacturing potential fundamentally recalibrating economics by enhancing yields, standardizing procedures, and reducing costs through scale. By thoroughly and sensitively examining regulatory frameworks, informed consent processes, risks and benefits, as well as financial considerations at both micro and macro levels, researchers, oversight boards and broader stakeholder networks can advance cell-based therapies towards delivering life-changing benefits for all communities. A multidisciplinary, conscientious approach balances progress against patient welfare. A combination of multiple strategies may help to overcome these limitations. For instance, gene-modified islets integrated within vascularized biomaterial implants or sequenced therapies have promising results to prime grafts in pro-regenerative environments before transplantation. Collaboration across disciplines offers hope that refined individualized therapies may eventually achieve durable insulin independence through functional pancreatic cell or tissue engraftment, not only for diabetes but also for chronic pancreatitis. Regarding, ongoing progresses in unraveling these barriers, cell replacement approaches have the potential to improve diabetes management.

## Conclusions

This review provides a comprehensive overview of the advances, challenges, and future directions in various cell-based therapeutic approaches for the treatment of diabetes. Significant progresses have been achieved in microencapsulation design, immunomodulation, tissue constructs, genetic and cellular reprogramming techniques, as well as initial clinical translation. However, the complete restoration of normoglycemia without the need for lifelong immunosuppression is still considered as a significant therapeutic challenge. Therefore, addressing the transplant environment of the hostile nature, developing minimally invasive delivery methods, and overcoming limitations in engraftment efficiency and longevity are crucial issues for the future researches. Through the sustained multidisciplinary efforts for the improvement of existing strategies and establishing novel paradigms, achieving durable insulin independence can be a realistic goal for all diabetic cases through the personalized cell replacement or regeneration.

**Table 1:** Summary of clinical trials evaluating various encapsulation and cellular delivery devices for the islet and stem cell transplantation in diabetic cases

Delivery system	Developer	Clinical trial ID	Description	Results
Encaptra device	ViaCyte	NCT02239354 NCT03163511 NCT03162926 NCT04678557	Semipermeable pouch containing pancreatic progenitor cells	The trial confirmed safety but hypoxia led to necrosis. Subsequent trials demonstrated increased C-peptide and 20% reduced required insulin for one year.
Semipermeable device	Semma/Vertex	NCT04786262	Houses differentiated stem cell-derived islet clusters	The trial improved glycemic control via portal vein infusion.
βAir device	Beta O_2_	NCT02064309	Alginate-PTFE membrane encapsulating islets	The trial showed small insulin amounts for up to 8 weeks but no insulin reduction, Oxygen reliance risks infection despite optimization efforts.
Cell Pouch device	Sernova	NCT03513939 NCT01652911	Pre-vascularized polypropylene chambers for islet transplantation	One trial showed transient C-peptide increase, Other trial reported sustained C-peptide for up to nine months.
Shielded Living Therapeutics spheres	Sigilon	NCT04541628	Cell clusters within alginate-TMTD coating	The hemophilia trial paused due to antibodies in highest doses, Safety concerns regarding TMTD coating remain.

**Table 2:** Summary of potential applications of additive manufacturing techniques for improving islet transplantation

Application	Objective	Approach	Benefits	Challenges
Implant Geometry	Enhance nutrient transport	3D printing scaffolds with defined Pore architecture	Increased surface area to volume ratio, precise microstructure control	Material restrictions of early am methods
Vascularization	Accelerate formation of vasculature	Bioprinting of vascular conduits and guides	Faster endothelial cell integration, optimized intra-device networks	Scaling construct size for clinical translation
Cell/Matrix Placement	Maintain optimal microenvironment	In Situ bioprinting of multiple components	Uniform composition, consistent presentation of signals	Refining multiple material deposition
Therapeutic Integration	Modulate Host/Graft responses	Printing of drug-loaded depots/gradients	Spatial control over cues, reduced toxicity risks	Characterizing release kinetics from complex designs

**Table 3:** Summary of gene therapy and cell therapy approaches studied for diabetes

Cell source	Gene/Protein Delivered	Vector	Study model	Outcomes	References
Liver cells	Insulin and glucokinase	Adeno-associated virus (AAV)	Dog with STZ-induced diabetes	Achieved normoglycemia without exogenous insulin long-term.	[119]
Liver	Pancreatic and duodenal homeobox 1 (PDX1)	AAV2	Humanized liver mouse model	Liver cells secreted insulin leading to glycemic control.	[120]
Hepatic cells	Neurogenin 3 (NGN3)	Adenovirus	Mice	Produced insulin and trans-differentiation of oval cells.	[121, 122]

## Data Availability

The datasets used and/or analyzed during the current study are available from the corresponding author on reasonable request.

## Abbreviations

ABS: Acrylonitrile butadiene styrene
APCs: Activate antigen-presenting cells
AAV: Adeno-associated virus
PDX1: Duodenal homeobox 1
EPIs: Engineered pseudo islets
ePTFE: Expanded polytetrafluoroethylene
ECM: Extracellular matrix
FBR: Foreign body response
FFF: Fused filament fabrication
GDM: Gestational diabetes mellitus
G6Pase: Glucose 6-phosphatase
IGFBP-1: Insulin-like growth factor binding protein-1
MSCs: Mesenchymal stem cells
NGN3: Neurogenin 3
OPTN: Organ Procurement and Transplantation Network
PEPCK: Phosphoenolpyruvate carboxykinase
PAEK: Polyaryletherketone
PCL: Polycaprolactone
PC: Polycarbonate
PEEK: Polyetheretherketone
PEI: Polyetherimide
PLA: Poly-lactic acid
PS: Polystyrene
SLA: Stereolithography
TPU: Thermoplastic polyurethane
T1D: Type 1 diabetes
T1DM: Type 1 diabetes mellitus
T2DM: Type 2 diabetes mellitus
UNOS: United Network for Organ Sharing
US: United States
VEGF: Vascular endothelial growth factor
